# Transvenous lead extraction of lumenless 3830 pacing lead in conduction system pacing: a single-center experience

**DOI:** 10.1007/s10840-023-01590-0

**Published:** 2023-06-27

**Authors:** Federico Migliore, Raimondo Pittorru, Manuel De Lazzari, Vincenzo Tarzia, Gianni Pastore, Lina Marcantoni, Domenico Catanzariti, Gino Gerosa, Francesco Zanon

**Affiliations:** 1 Department of Cardiac, Thoracic, Vascular Sciences and Public Health, University of Padova, Via N. Giustiniani 2, Padova, Italy; 2 Arrhythmia and Electrophysiology Unit, Division of Cardiology, Santa Maria Della Misericordia General Hospital, Rovigo, Italy; 3 Division of Cardiology, Santa Maria del Carmine Hospital, Rovereto, TN Italy

**Keywords:** Conduction system pacing, Lumenless 3830 pacing lead, Transvenous lead extraction

## Abstract

**Background:**

The Medtronic SelectSecure Model 3830 lumenless lead (Medtronic, Inc., Minneapolis, MN) is commonly used for conduction system pacing (CSP). However, with this increased use, the potential need for transvenous lead extraction (TLE) also will increase. While extraction of endocardial 3830 leads is rather well described especially in pediatric and adult congenital heart disease population, there is very limited data on extraction of CSP leads. In the present study, we reported our preliminary experience on TLE of CSP leads and provided technical considerations.

**Methods:**

The study population comprised 6 consecutive patients (67% male; mean age 70 ± 22 years) with CSP leads (3830 leads), including left bundle branch pacing (LBBP) lead (*n* = 3) and His pacing lead (*n* = 3) undergoing TLE. Overall target leads were 17. The mean implant duration time of CSP leads was 97 ± 90 months [range 8–193).

**Results:**

Manual traction was successful in 2 cases and mechanical extraction tools were required in the remaining cases. Sixteen leads (94%) were completely extracted, whereas incomplete removal was observed in one lead (6%) among 1 patient. Of note, in the only lead incompletely removed, we observed retention of < 1-cm remnant of lead material consisting of the screw of 3830 LBBP lead into the interventricular septum. No failure of lead extraction was reported and no major complications occurred.

**Conclusions:**

Our findings demonstrated that at an experienced center the success of TLE of chronically implanted CSP leads is high in the absence of major complications also when mechanical extraction tools are needed.

## Introduction

**Fig. 1:**
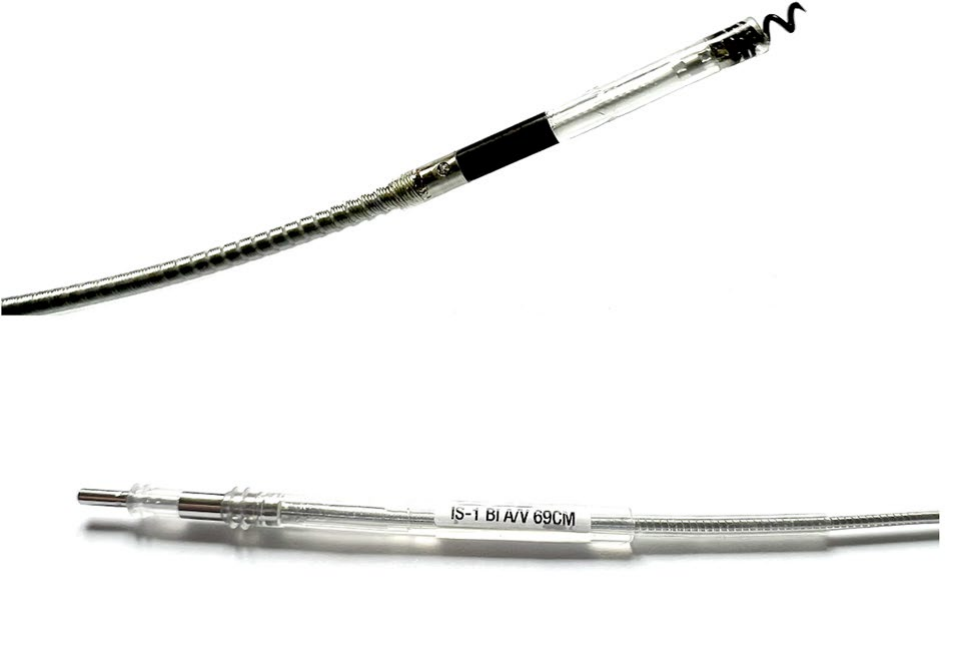
Medtronic 3830 lead

Conduction system pacing (CSP) has emerged as a more physiological alternative to right ventricular (RV) pacing and is also being used in selected cases for cardiac resynchronization therapy (CRT) [1]. His bundle pacing (HBP) was first introduced over two decades ago and its use has risen over the last 5 years with the advent of tools which have facilitated implantation [1–3]. However, it has some limitations, such as operational difficulty and higher pacing thresholds [1–3]. Thus, recently, left bundle branch pacing (LBBP) via a transventricular-septal approach has emerged as an alternative physiologic pacing with a low, stable pacing capture threshold and relatively narrow QRS duration due to fast left ventricular activation and direct excitation of the diseased LBB [1, 4]. Implantation of CSP has risen dramatically and data on its efficacy and safety are growing fast [1–5]. The Medtronic SelectSecure Model 3830 lumenless lead (Medtronic, Inc., Minneapolis, MN, Fig. 1) is commonly used for CSP [1–5]. However, with this increased use, the potential need for transvenous lead extraction (TLE) also will increase. Lumenless lead construction of the 3830 lead requires an understanding of both applicable tensile forces and lead preparation techniques that can influence consistent extraction [6–8]. Although the potential benefit of 3830 lead in ease of extraction has been reported in the literature, the data are limited [9, 10] and only few reports exist on extraction of CSP, especially on LBBP and when powered sheaths are required [11–15]. In the present study, we reported our preliminary experience on TLE of CSP leads and provided technical considerations. Moreover, the goal is to provide physicians with a potential approach for safe and successful extraction of CSP using 3830 leads.

## Methods

The study population comprised of 6 consecutive patients with CSP leads undergoing TLE in our center (Department of Cardiac, Thoracic, Vascular Sciences and Public Health, University of Padova, Italy) from October 2020 to April 2023. The study was conducted in compliance with the principles outlined in the Declaration of Helsinki and approved by the local medical ethics committee. The device manufacturers did not sponsor or influence the study in any way.

### Data acquisition

Demographic, historical, and procedural data were collected for each patient. The underlying type, number, and fixation modality of each lead were included among the recorded variables. Indications for TLE were classified as infection (local or systemic), lead dysfunction, pre-existing system upgrade, or other.

### Extraction procedure

**Fig. 2:**
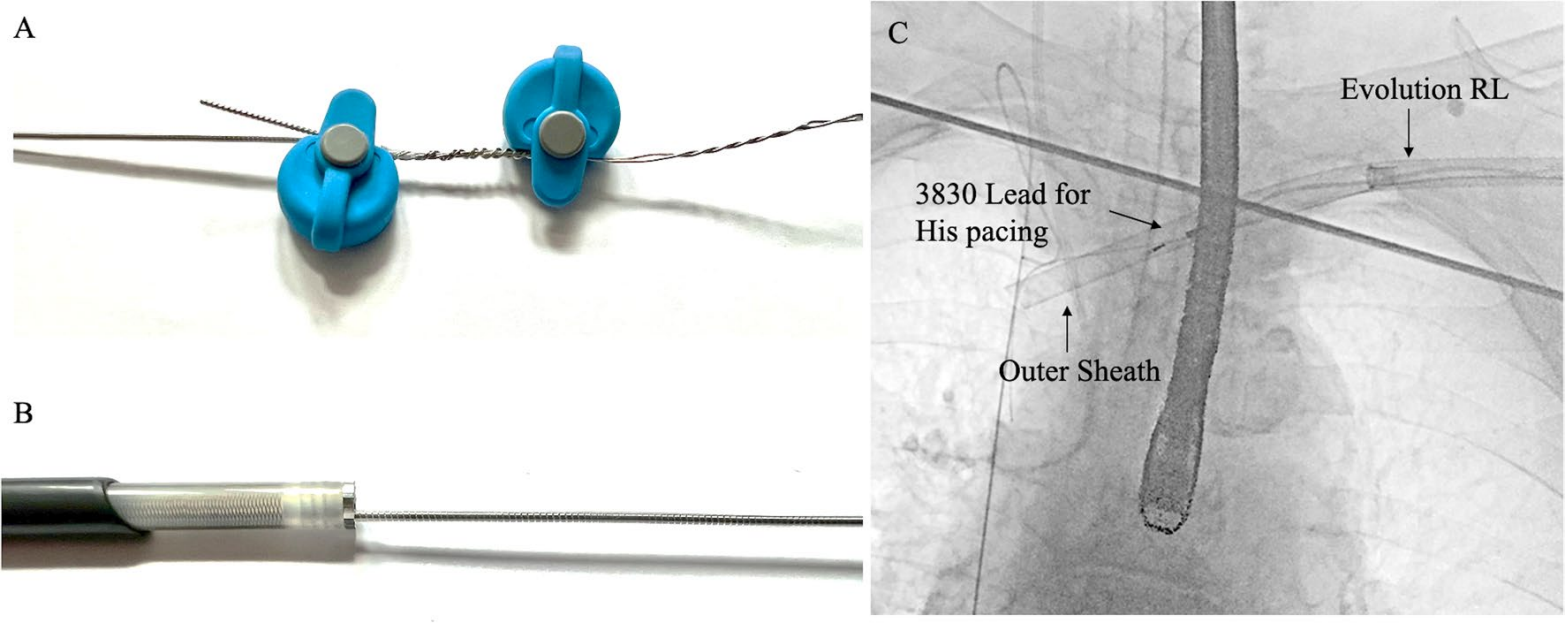
Lead preparation technique (**A**). Given the lumenless design of the 3830 lead, a Bulldog lead extender (Cook Medical) was applied with the addition of a One-Tie compression coil (Cook Medical) around the lead extender unit and on the outer insulation. Evolution RL rotational sheath was used when manual traction was ineffective in chronically implanted leads (**B**, **C**)

All procedures were performed by electrophysiologists experienced in lead extractions in the operating room under general anesthesia with continuous electrocardiographic and arterial blood pressure monitoring using transesophageal echocardiography (TE) guidance. Standby cardiac surgery including the availability of extracorporeal circulation and a perfusionist for the treatment of emergency complications was always available, and so was a stiff guidewire from the right femoral vein to the right internal jugular vein for potential use of the bridge occlusion balloon (Philips-Spectranetics, Colorado Springs, CO, USA) in cases of vascular lacerations. In patients dependent on bradycardia support, a temporary pacemaker (PM) was inserted through the femoral vein. Angiography of the subclavian vein was performed before the TLE, which was performed using a stepwise systematic approach [16]. The leads were disconnected from the header and prepared for extraction. In case of traditional leads, the active fixation mechanism was retracted and manual traction was attempted. If this was unsuccessful, manual traction was attempted again using a locking stylet (Cook Medical, Bloomington, IN, USA) and One-Tie compression coil (Cook Medical). In case of the 3830 lead, gentle manual traction and counterclockwise rotations were attempted. If this was unsuccessful, given the lumenless design of the 3830 lead, the connector was cut, and a Bulldog lead extender (BDLE), by Cook Medical, Bloomington, was applied with the addition of a One-Tie compression coil (Cook Medical) around the lead extender unit (Fig. 2A). This reinforces the lead assembly and reduces the risk of lead fracture at the interface where the lead extender causes bending of the lead [9]. According to our experience, extraction of CSP leads was attempted after removing conventional leads in order to improve adherence and thus avoid increased strain on the stylet-less 3830 lead. When manual traction was ineffective, fibrous adhesions surrounding the lead were dissected using the Evolution RL rotational sheaths (Fig. 2B, C) with available extraction tools (Evolution Shortie RL, One-Tie Compression Coil, and SteadySheath Evolution tissue stabilisation sheath; Cook Medical) in all procedures as previously reported [16]. A SteadySheath Evolution tissue stabilization sheath was used in cases of extensive scarring or calcification limiting the advancement of Evolution RL after several attempts and adjustments. In patients with PM dependency and infection, a new active-fixation RV pacing lead was inserted at the ipsilateral side, or at the internal jugular vein, contralateral to the side of the infection. The lead was sutured to the patient’s skin with non-resorbable sutures, and the external section of the lead was then connected to a permanent PM pulse generator. Device reimplantation of the permanent device and removal of the temporal RV lead were then performed in an infection-free interval. All patients were monitored for procedure-related complications at the time of extraction, during their hospital stay, and at scheduled follow-up appointments. Patients were followed up with serial outpatient evaluations or with telephone interviews to determine whether they experienced any adverse event.

### Definitions

Success, failure, and complications were defined according to the definitions of the 2017 Heart Rhythm Society and the 2018 European Heart Rhythm Association expert consensus [17, 18].

### Statistical analysis

Continuous variables are expressed as mean ± standard deviation or median values and 25^th^ and 75^th^ percentiles. Categorical variables are presented as actual numbers and frequencies. The Shapiro–Wilk *W* test was used to assess the normality of continuous variables.

## Results

**Fig. 3:**
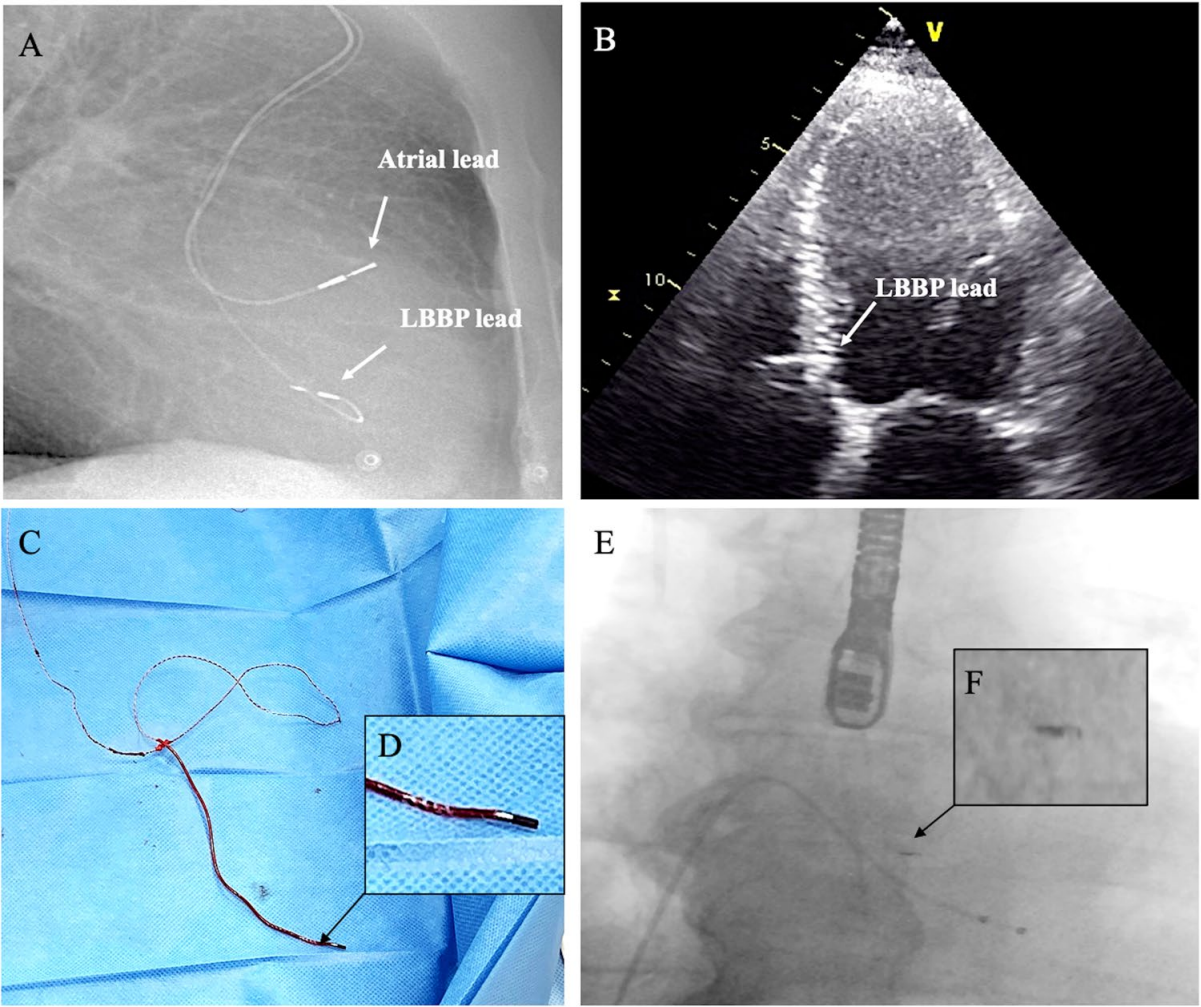
Patient no. 6. Lateral chest X-ray view showing the atrial lead and the LBBP lead (**A**). Four-chamber echocardiography view showing the LBBP lead deep into the interventricular septum (**B**). Removed 3830 lead (**C**). Note the absence of the screw after lead extraction (**D**). Fluoroscopy view immediately after removal of the leads under TE guidance (**E**) showing the retention of the screw into the interventricular septum (**F**). LBBP, left bundle branch pacing

The study population comprised 6 patients (67% male (*n* = 4); mean age 70 ± 22 years, median 79 (66–83)) with 17 target leads. Patients, CSP extracted leads characteristics, and outcomes are reported in Table 1. Indications for TLE included infection in 4 cases (66%; local infection 33%, systemic infection 33%), lead malfunction and upgrading to implantable cardio verter defibrillator (ICD) in 1 case (17%), and, finally, lead-related severe tricuspid valve regurgitation in 1 case (17%). Of the target extracted leads, 6 (35%) were right atrial leads, 3 (18%) were RV pacing leads, 2 (12%) were ICD leads (2 single-coil), and 6 (35%) were CSP leads (LBBP *n* = 3; s bundle HBP *n* = 3). The mean implant duration time of CSP leads was 97 ± 90 months [range 8–193; median 99 months (14–174 months)]. One extracted His lead was previously abandoned for failure (patient no. 5, see Table 1). Occlusion of the left subclavian vein was observed in 1 patient (16%). No recalled leads were reported. Manual traction was successful in 2 cases and mechanical extraction tools (Evolution RL sheaths, Cook Medical, Bloomington, IN, USA) were required in the remaining cases (Table 1). Sixteen leads (94%) were completely extracted, whereas incomplete removal was observed in one lead (6%) among 1 patient (16%). Of note, in the only lead incompletely removed, we observed retention of a < 1-cm remnant of lead material consisting of the screw of 3830 lead (patient no. 6, see Table 1) without embolization. In this case, before the lead extraction procedure, angiography revealed a subocclusion of the left subclavian vein. We extracted the LBB pacing lead using the Evolution RL rotational sheath (11F). The sheath was gently advanced over the lead until the right atrium, where we were able to remove the lead but the tip remained adherent within the interventricular septum (Fig. 3). No failure of lead extraction was reported. No additional use of tools via the femoral or jugular approach was required. Complete procedural success rate, clinical success rate, and lead removal with clinical success rate were 83% (5/6), 100% (6/6), and 100% (17/17), respectively. Four patients (67%) were reimplanted with a leadless PM (*n* = 1), transvenous PM (*n* = 1), transvenous ICD (*n* = 1), and epicardial leads (*n* = 1). Two patients were not reimplanted based on clinical decision. Subsequently, one of them successfully underwent heart transplant.

**Table 1:** Patients, extracted lead characteristics, and outcomes

	Sex	Age	Indication to pacing	Type of CSP	Type of device	No. of target leads	Dwell time of CSP lead	Indication to TLE	Type of extraction technique for CSP lead	Retention of remnant of lead
Patient no. 1	Female	28	CRT	LBB	CRT-D	3	10 months	Lead-related severe tricuspid valve regurgitation	Manual traction	None
Patient no. 2	Male	83	SSS, LBBB	His bundle	Dual-chamber PM with backup RV lead	3	14 years and 3 months	Local infection	Evolution RL 11F, 13 F, SteadySheath 13 F	None
Patient no. 3	Male	63	CRT	LBB	CRT-D	3	8 months	Systemic infection	Manual traction	None
Patient no. 4	Male	91	AV block	His bundle	Dual-chamber PM with backup RV lead	3	14 years and 6 months	Systemic infection	Evolution RL 11 F	None
Patient no. 5	Female	84	SSS, AV block	His bundle	Dual-chamber PM and His abandoned lead for failure	3	16 years and 1 month	Local infection	Evolution RL 9F, 11F	None
Patient no. 6	Male	75	AV block	LBB	Dual-chamber PM	2	2 years and 4 months	Lead failure and upgrading to ICD	Evolution RL 11F	The screw into the interventricular septum

*AV*, atrio-ventricular; *CRT*, cardiac resynchronization therapy; *CRT-D*, cardiac resynchronization therapy-defibrillator; *CRT-P*, cardiac resynchronization therapy-pacemaker; *ICD*, implantable cardioverter defibrillator; *LBB*, left bundle brunch; *PM*, pacemaker; *RV*, right ventricular; *SSS*, sick-sinus syndrome

### Procedural complications and follow-up

No major complications occurred. One minor complication was reported, consisting on femoral artero-venous fistulae with need for treatment. No damage of the conduction system was observed and no evidence of interventricular septal defect in case of LBB pacing was detected after TLE under TE guidance. During a mean time follow-up of 15 ± 13 months [median 15 (3–26)], only one patient died because of urosepsis. No procedure-related mortality occurred.

In patients who underwent LBB pacing lead removal for lead-related tricuspid valve regurgitation (patient no. 1, see Table 1), post-procedural TE revealed a moderate TR and no evidence of interventricular septal defect.

## Discussion

### Lumenless 3830 lead

The 3830 lead has several features which make it distinct from standard pacing leads [6–8]. It is a bipolar, narrow-body lead with a diameter of 4.1 French. An inner conductor cable for the tip electrode is covered with an inner silicone insulation. The outer ring conductor coil wraps around the silicone insulation and is itself covered by an outer polyurethane layer [8]. The lead is lumenless and actively fixated with a non-retractable exposed helix that is attached to a steroid-eluting tip (Fig. 1). It is implanted using either a steerable or pre-formed sheath depending on the site of delivery. The 3830 was initially developed for use in the pediatric population, and currently, the advent of CSP has seen a significant increase in its use. Lumenless pacing leads for CSP compared with Stylet-driven pacing leads (SDLs) present potential advantages and potential disadvantages [1]. Potential advantages include [1] smaller lead body (4.1Fr) might result in less interference with septal kinetics and less risk for collateral damage to septal vessels or septal injury; [2] isodiametric lead design facilitates septal penetration; [3] helix is robust and retraction will not occur; and [4] in case of LBBP failure, HBP as bail out might be easier than with SDLs [1]. Potential disadvantages include [1] absence of stylet may result in less stiffness and torque ability of the lead; [2] smaller lead body can result in less grip on the lead when rotating the lead; [3] delivery sheaths with smaller diameters might be less supportive and more prone to kinking [1].

### Extraction of lumenless 3830 lead

Although the 3830 leads could be removed with manual traction alone in most cases compared to the conventional leads [10], the features of the 3830 lead may impact upon the technical aspects of a TLE. First, the use of locking stylets is not possible, raising the need for extraction tools such as lead extenders and compression ties especially in chronically implanted lumenless leads when extraction tools are needed [6, 7]. In an extraction, a mechanical or powered sheath is advanced over the lead to free the lead to remove scar attachments. Accomplishing feasible extraction requires the lead to act as a stable extraction rail for the extraction tool to track. In leads with stylet lumens, that rail is composed of all the components of the lead body and the selected locking stylet that is deployed and locked within the inner conductor lumen. Therefore, proper lead preparation is a critical aspect to providing appropriate rail strength and maintaining lead integrity when extracting the 3830 lead. Recently, Vatterott et al. [6] demonstrated that preservation of the proximal connector end of lumenless leads provided the most consistent and best ability to tolerate extraction loading forces compared to cutting of the connector. However, no statistical significance of rail strengths was observed between the cut lead method with BDLE and One-Tie Compression Coil and the retained connector method [6]. Of note, this study was not performed in humans and it was not designed to provide specific information on extraction of CSP. In our experience by using the BDLE and One-Tie Compression Coil around the lead extender unit (Fig. 2), we observed a good lead rail strength. Second, the presence of a non-retractable helix raises questions about the possible risks of myocardial avulsion when extracting leads with an increased dwell time potentially complicating TLE procedure [6, 7].

### Transvenous lead extraction in conduction system pacing

While extraction of endocardial 3830 leads is rather well described especially in pediatric and adult congenital heart disease population, there is very limited data on extraction of CSP leads. Evidence is limited to retrospective datasets with relatively short mean dwell time [11] and single case reports [12–14]. Concerns remain about potential injury to the conduction system during extraction and the lack of lumen for placing a locking stylet for TLE when powered sheaths are required in chronically implanted lumenless leads. Moreover, the presence of more leads including conventional leads (e.g., atrial/RV pacing/ICD leads) and CSP leads represents a further possible concern for the removal sequence of the leads [6, 8, 13, 14].

Vijayaraman et al. [11] in a retrospective study of 30 patients who underwent TLE of HBP leads, with a mean dwell time of 25 ± 18 months, reported removal of HBP leads was successful in 8 of 8 patients (100%) with ≤ 12-month duration and 21 of 22 patients (95%) with > 12-month duration. Extraction tools were used in 4 patients (13%), while manual traction was successful in the remaining patients and one extraction was unsuccessful.

With the increased interest in LBBP, there have also been questions raised about TLE of these systems, given the deep septal location and potential for myocardial avulsion and iatrogenic ventricular septal defects. To date, there have been 3 case reports of LBBP lead extraction with manual traction, with all leads being ≤ 2 years old, one from our experience [12, 14, 19]. No complications were observed. However, it should be noted that there is concern about partial extraction in LBBP systems because LBBP involves boring the lead into the interventricular septum, thus creating a fulcrum, and consequently a stress point at that fulcrum leading to lead fractures and partial extractions [1, 8, 20].

Our experience, albeit limited, demonstrated the safety and efficacy of TLE of CSP leads, including HBP and LBBP using mechanical extraction tools when manual traction is unsuccessful. However, it should be noted that in cases of prolonged dwell time, there is the potential risk of incomplete extractions due to the tip into the interventricular septum (see Table 1, patient no. 6). Moreover, the presence of more leads represents a further peculiarity of our case series raising others possible concerns for the removal sequence of the leads. We believe the tensile strength of the 3830 lead allows the use of powered tools without the need for a locking stylet due to the lead design of the lead. However, we suggest to perform the CSP leads extraction after removing other leads with severe adherence, avoiding increased strain on the styletless lead, as suggested also by others [6]. Although the 3830 is a thin lead, in case of prolonged dwell time and in the presence of more leads, according to our experience we suggest to use larger sheaths (Evolution RL 11F and 13F) because fibrosis increases the diameter of the lead body, the friction of passing the extraction sheath over the lead, and the stress of the tensile properties of the lead. Using a larger sheath not only may increase the stiffness but also may ease the passing of the sheath over the lead body [6]. No damage of the leads was observed with the Evolution RL at any angle because the cutting surface is external in the bench study by Vatterott et al. [6]. While experience is limited, in our experience it seems like there is no need to advance to the tip. However, much larger studies with longer residence times could provide us with more evidence. But it should be remembered that in extraction procedures each case is unpredictable. Last but not least, we recommend to perform the procedure in high-volume centers with experienced operators and different available tools.

While the 3830 lead is the most widely used lead for the delivery of CSP, there have been reports of LBBP using SDLs [1]. Cases of leads with an extendable helix and fracture of the helix rotating mechanism during lead extraction have been described [21, 22].

### Limitations

This is a preliminary single-center experience with only six cases. However, currently, evidence is very limited. When manual traction was unsuccessful, we used exclusively bidirectional mechanical TLE with the Evolution RL system. No direct comparison has been made between the Evolution RL system and other currently used techniques for TLE (ThightRail or Laser, Philips). Hence, no conclusion can be drawn regarding the comparative efficacy or safety of mechanical versus laser sheaths. TLE procedures in our department were performed by a team experienced using bidirectional mechanical TLE sheaths; therefore, our results may not be widely applicable in less experienced centers. Finally, the number of participants included in our study was relatively small.

## Conclusions

Our results demonstrated that in an experienced center, the extraction success of CSP leads with short and medium implant durations is high in the absence of major complications even when mechanical extraction tools are required. Although limited in number of cases, our preliminary experience provides a framework for future larger studies.

## Data Availability

Data available on request from the authors.
